# Metabolically healthy obesity and depressive symptoms: 16-year follow-up of the Gazel cohort study

**DOI:** 10.1371/journal.pone.0174678

**Published:** 2017-04-06

**Authors:** Guy-Marino Hinnouho, Archana Singh-Manoux, Alice Gueguen, Joane Matta, Cedric Lemogne, Marcel Goldberg, Marie Zins, Sébastien Czernichow

**Affiliations:** 1 Inserm, UMS 011, Villejuif, France; 2 Inserm, U1018, Centre for Research in Epidemiology and Population Health, Villejuif, France; 3 University Versailles St-Quentin en Yvelines, Versailles, France; 4 Department of Epidemiology and Public Health, University College London, London, United Kingdom; 5 Department of Nutrition, Holy Spirit University, Jounieh, Lebanon; 6 Université Paris Descartes, Sorbonne Paris Cité, Faculté de médecine, Paris, France; 7 AP-HP, Hôpitaux Universitaires Paris Ouest, Service de Psychiatrie de l'adulte et du sujet âgé, Paris, France; 8 Inserm U894, Centre Psychiatrie et Neurosciences, Paris, France; 9 Department of Nutrition, European Hospital Georges Pompidou, Paris, France; Tulane University School of Public Health and Tropical Medicine, UNITED STATES

## Abstract

**Aims:**

The health correlates of the metabolically healthy obese (MHO) phenotype, particularly in relation to depressive symptoms remains unclear. Accordingly, we examined the risk of depressive symptoms in this phenotype using a 16-year follow-up prospective study.

**Methods:**

A sample of 14 475 participants (75% men), aged 44–59 years in 1996, was drawn from the Gazel cohort. Obesity was defined as body mass index (BMI) ≥ 30 kg/m^2^ and metabolic health as having none of the self-reported following cardiovascular risk factors: hypertension, type 2 diabetes and dyslipidemia. Depressive symptoms were assessed by the Center for Epidemiologic Studies Depression (CES-D) scale in 1996, 1999, 2002, 2005, 2008 and 2012. Generalized Estimating Equations (GEE) were used to estimate the risk of depressive symptoms during a follow-up of 16 years.

**Results:**

In multivariate analyses, metabolically unhealthy normal weight [Odds Ratio (OR) = 1.37; 95% Confidence Interval (CI): 1.25–1.51], overweight [1.44 (1.31–1.59)] and obese [1.30 (1.10–1.54)] but not MHO participants [1.04 (0.81–1.32)] had higher risk of depressive symptoms at the start of follow-up compared to metabolically healthy normal weight individuals. Depressive symptoms decreased over time in metabolically healthy normal weight individuals [0.52 (0.50–0.55)], this decrease was less marked only in metabolically unhealthy obese participants [1.22 (1.07–1.40)]. Compared to MHO participants, metabolically unhealthy obese individuals were at increased risk of depression at the start of follow-up, but with a similar reduction of this risk over time.

**Conclusion:**

Poor metabolic health, irrespective of BMI was associated with greater depressive symptoms at the start of follow-up, whereas a poorer course of depressive symptoms over time was observed only in those with both obesity and poor metabolic health.

## Introduction

Obesity is often accompanied by the metabolic syndrome [1], which is a cluster of cardiometabolic abnormalities such as raised fasting blood glucose, high blood pressure and dyslipidemia. However, not all obese persons have metabolic abnormalities and such individuals constitute the metabolically healthy obese (MHO) phenotype [2] with some evidence that the impact of obesity on health can be avoided in these individuals [3]. There is considerable interest in the health correlates of this phenotype [4–8] although results are not consistent across studies or the health outcomes examined.

Obesity and depression are two major public health concerns worldwide [9], both confer an increased risk for type 2 diabetes [10] and cardiovascular diseases [11]. Some studies show obesity to be associated with an increased risk of depressive symptoms [12,13], others suggest a bidirectional [14,15] or no association between the two [16]. Metabolic syndrome appears to be associated with depression independently of obesity [17]. Assessment of the association between MHO phenotype and depressive symptoms is likely to shed light on the relationships between obesity and depression. To our knowledge, only two studies have examined the association between MHO phenotype and depressive symptoms [18,19] with one showing no increased risk of depressive symptoms in MHO individuals followed for 2 years compared with healthy non-obese individuals [18]. However, in the second report, a pooled analysis of eight cross-sectional studies, obese individuals with a favorable metabolic profile had a slightly increased risk of depressive symptoms compared with healthy non-obese persons [19].

Prospective studies with long follow-ups that have examined the risk of depression in MHO persons are sorely lacking. In addition, in the two previous studies, assessments of depressive symptoms were undertaken at only one [19] or two time points [18]. Accordingly, the aim of the present study was to examine the association between obesity with or without cardiometabolic abnormalities and depressive symptoms during a follow-up period of 16 years.

## Materials and methods

### Study population

As described in detail elsewhere [20], data were drawn from the Gazel cohort, an on-going epidemiological study set up in 1989 on employees of France’s national electricity and gas company (EDF-GDF, Electricité de France-Gaz de France). At the start of follow-up, a total of 20 625 employees (15 011 male and 5 614 female) aged 35–50 years old gave written informed consent to participate. In January of each year since 1989, participants have completed a self-administrated questionnaire on their lifestyle, health, and occupational situation. In addition to the annual questionnaire, a comprehensive update includes data from the human resources department, the firm’s medical insurance program and the department of occupational medicine. The attrition rate in the study is low (0.5% in 2008 at the 20-year follow-up), approximately 75% of participants respond to the study questionnaire every year [21]. For the present analysis, exposure measures were drawn from the period between 1990 and 1996 and outcome measures between 1996 and 2012.

The study was approved by the French authority for data confidentiality (Commission Nationale Informatique et Libertés) and by the Ethics Evaluation Committee of the Institut National de la Santé et de la Recherche Médicale (INSERM).

The period 1990–1996 constitutes the measurement period of the exposure: Body Mass Index (BMI) and metabolic status.

**BMI** was calculated from self-reported weight and height. Height was self-reported in 1990 and data on weight were collected through annual questionnaires from 1990 to 1996. All participants who reported their weight at least once over this period were included in the analysis. Body mass index (BMI) was calculated by dividing weight (in kilograms) by height (in meters squared) and categorized using the WHO classification [22]: <18.5 kg/m^2^ (underweight), 18.5–24.9 kg/m^2^(standard weight), 25–29.9 kg/m^2^ (overweight) and ≥30 kg/m^2^ (obese), with the under 18.5 category (N = 192) removed from the analysis. Mean BMI over this period was used in the analysis.

**Metabolic status** was defined as reported physician diagnosis and treatment of hypertension, type 2 diabetes, or hypercholesterolemia over the period 1990 to 1996. These conditions were assessed using the following question "Do you suffer or have you suffered from these disorders during the previous 12 months?”. One positive response over the exposure window of 1990 and 1996 led participants to be classified as being metabolically unhealthy and no positive response over the period as metabolically healthy. We used this definition alongside data on BMI to create six phenotypes: metabolically healthy-normal weight (MH-NW), metabolically healthy-overweight (MH-OW), metabolically healthy obese (MHO), metabolically unhealthy-normal weight (MU-NW), metabolically unhealthy-overweight (MU-OW) and metabolically unhealthy obese (MUO).

### Covariates

Analyses were adjusted for age, sex, marital status, occupational position, physical activity, fruit and vegetable consumption, alcohol intake and smoking status. Data on age and sex was obtained from company human resources. As described in detail previously [20], the measure of occupational position was taken from the employer’s records of grade of employment at age 35 (representative of mid-career status) and categorized as executives, intermediate profession, employees and manual workers. Marital status (single or married/cohabiting), physical activity (competition level, regular but not competition level, occasionally, and none), alcohol intake (none, moderate: 1–21 units/week in men and 1–14 in women, heavy: ≥ 22 units/w in men and ≥ 15 in women), smoking status (non-smokers, current smokers and ex-smokers) and fruit and vegetable consumption (< 1, 1–2 and > 2 times/week) were self-reported in 1990.

**Depressive symptoms** were measured in 1996, 1999, 2002, 2005, 2008 and 2012 using the Center for Epidemiologic Studies Depression (CES-D) scale [23]. This 20-item questionnaire evaluates symptoms and behaviors characteristic of depressive disorders and has been designed for use in community studies. The CESD asks participants how often they have experienced specific symptoms during the previous week (e.g., “I felt depressed”; “I felt everything I did was an effort”; “My sleep was restless”). Responses range from 0 “hardly ever” to 3 “most of the time”. The widely used threshold of a score ≥ 16 out of 60 designated presence of depressive symptoms, it has also been shown to identify individuals at risk for clinically significant depression [23].

### Statistical analysis

The characteristics of the participants at the start of the follow-up are presented as percentage or mean (SD) when appropriate, by metabolic status and as a function of BMI categories. We used chi-square test to test for differences between groups in baseline characteristics for dichotomous measures and ANOVA for continuous variables. Over the follow-up period, episodes of depressive symptoms (i.e. having a CES-D score ≥ 16) were categorised as 0 (no episodes) and 1 (at least one episode).

The associations between BMI, metabolic status, BMI-metabolic status phenotypes and depressive symptoms were examined using Generalized Estimating Equations (GEE) models in order to account for the correlation between repeated observations on the same participant [24]. The interaction terms between sex and BMI-metabolic status phenotypes (p for interaction > 0.27), between sex, time and BMI-metabolic status phenotypes (p for interaction > 0.15) revealed no differences, allowing us to combine men and women in the analyses. The longitudinal estimates were modelled to reflect change over a 10-year period. The interaction term between time and BMI-metabolic status phenotype was used to estimate 10-year change of depression across subgroups.

In the first set of analyses, the metabolically healthy-normal weight phenotype was used as the reference category. Odds-ratio (OR) and 95% confidence intervals (95% CI) were adjusted for age, sex, occupational position, marital status, smoking status, alcohol intake, physical activity, fruit and vegetable consumption. In a second set of analyses, we compared the risk of depressive symptoms as a function of metabolic health status in each BMI category. The metabolically healthy group, within each BMI category, was the reference in these analyses.

In sensitivity analyses, episodes of depressive symptoms were classified as: 0 (no episodes) 1 (1–3 episodes) and 2 (4–6 episodes); these analyses were undertaken using multinomial logistic regression models. In another set of sensitivity analyses, a higher threshold (CES-D ≥ 23) was used to define the presence of depressive symptoms in order to test the robustness of our results. All analyses were undertaken using STATA 11 (StataCorp. College Station, TX, USA). Reported p values are 2-tailed and p values <0.05 were considered to be statistically significant.

## Results

Of the 20 625 participants recruited to the study in 1989, 6 150 were excluded for one or more of the following reasons: BMI<18.5 kg/m^2^ (n = 192), missing data on BMI-metabolic status phenotype (n = 3 100); a further 3 266 persons did not have complete data on CESD over the follow-up. The final sample consisted of 14 475 participants (10 814 men and 3 661 women). Compared to those excluded, participants included in the analyses were older (43.9 years vs. 43.5 years), more likely to be men (74.7% vs. 68.2%), married or cohabiting (86.7% vs. 81.7%), physically active (67.4% vs. 60.7%), and to have a higher occupational position (15.1% vs. 9.7%), all p<0.01.

Baseline characteristics of participants included in the analysis by metabolic status and BMI categories are presented in Table 1. Of these participants, 57.0% (n = 8244) were metabolically healthy and 6.2% (n = 902) were obese. The metabolically healthy obese phenotype represented 2.0% (n = 298) of the total analytic sample and 33.0% of the obese population. They were younger, with moderate alcohol consumption and high socioeconomic status compared to metabolically unhealthy obese. The prevalence of depressive symptoms in the study population was 32.4%, 31.4%, 26.2%, 23.3%, 22.5% and 19.3% respectively in 1996, 1999, 2002, 2005, 2008 and 2012. A total of 52.6% of participants reported depressive symptoms at least once during follow-up.

**Table 1:** Sample characteristics at baseline as a function of metabolic health status and Body Mass Index (1996).

	Metabolically Healthy (N = 8244)			Metabolically Unhealthy (N = 6231)		
	Normal weight	Overweight	Obese	Normal weight	Overweight	Obese
N (%)	4934(34.1)	3012(20.8)	298(2.0)	2556(17.7)	3071(21.2	604(4.2)
Age,years(SD)	43.1(3.7)	44.2(3.2)	43.9(3.4)	44.2(3.5)	44.7(3.0)	44.5(3.0)
Male Sex (%)	59.5	88.0	78.9	67.7	89.9	82.8
Marital status (%), Married or cohabiting	84.0	89.9	83.3	84.6	90.2	86.1
Socioeconomic status(%), Executives	15.6	15.4	10.4	16.6	14.5	9.2
Smoking(%), Non-smokers	23.4	26.4	26.3	25.3	27.1	27.5
Alcohol consumption, Moderate	90.0	87.5	86.2	87.7	84.3	80.0
Fruits consumption, 1–2times/week	11.7	13.7	14.8	12.7	13.5	15.6
Vegetables consumption, 1–2times/week	32.7	36.6	35.0	32.5	34.1	31.6
Physical activity,occasionnally	29.7	33.4	30.9	31.2	34.3	24.8
Diabetes	-	-	-	6.9	8.0	21.7
Hypertension	-	-	-	38.2	46.0	65.9
Hypercholesterolemia	-	-	-	72.1	74.7	69.9

Values are mean (SD) unless otherwise indicated

Table 2 shows the associations of BMI-metabolic status phenotypes with depressive symptoms. At the start of the follow-up in 1996, compared with metabolically healthy normal weight participants, metabolically unhealthy normal weight (OR = 1.37, CI 95% 1.25 to 1.51), overweight (OR = 1.44, CI 95% 1.31 to 1.59) and obese individuals (OR = 1.30, CI 95% 1.10 to 1.54) but not MHO individuals (OR = 1.04, 95% CI: 0.81 to 1.32) had higher risk of depressive symptoms. Depression rates decreased over the follow-up in metabolically healthy normal weight individuals; the OR of change over 10 years (95% CI) was 0.52 (0.50–0.55). However, the decrease in depression risk over time was less marked only in obese participants with metabolic abnormalities (OR = 1.22, 95%: 1.07 to 1.40).

**Table 2:** The association of BMI-metabolic status^†^ phenotypes (1990–96) with depressive symptoms (1996/2012).

	At baseline OR (95%CI)	10-year Change over the follow-up OR (95% CI)
**Time**	-	0.52(0.50–0.55)
**BMI-metabolic health status**		
Metabolically Healthy Normal Weight	1 (ref)	1 (ref)
Metabolically Healthy Overweight	0.99 (0.90–1.09)	1.01 (0.93–1.10)
Metabolically Healthy Obese	1.04 (0.81–1.32)	1.07 (0.88–1.31)
Metabolically Unhealthy Normal Weight	1.37 (1.25–1.51)	0.97 (0.90–1.05)
Metabolically Unhealthy Overweight	1.44 (1.31–1.59)	1.00 (0.92–1.08)
Metabolically Unhealthy Obese	1.30 (1.10–1.54)	1.22 (1.07–1.40)

OR: Odds-ratio; CI: Confidence Interval. Analyses adjusted for age, sex, socioeconomic status, marital status, physical activity, smoking status, alcohol, fruit and vegetable consumption.

^†^Defined as reported physician diagnosis and treatment of any of these three conditions: hypertension, type 2 diabetes and hypercholesterolemia.

Table 3 depicts the association between metabolic status and depressive symptoms in analysis stratified by BMI category; the metabolically healthy group was the reference within each strata of BMI. Regardless of BMI category, the metabolically unhealthy individuals had an increased risk of depression at the start of the follow-up in 1996 compared with their metabolically healthy counterparts. However, the decrease in depression risk over time was similar between metabolically healthy and unhealthy individuals in each strata of BMI.

**Table 3:** The association of metabolic health status^†^ (1990/96) with depressive symptoms (1996/2012) in analyses stratified by BMI categories.

	At baseline OR (95%CI)	10-year Change over the follow-up OR (95%CI)
**Normal weight**		
Metabolically Healthy	1	1
Metabolically Unhealthy	1.37 (1.24–1.51)	0.97 (0.90–1.05)
**Overweight**		
Metabolically Healthy	1	1
Metabolically Unhealthy	1.47 (1.32–1.63)	0.98 (0.90–1.07)
**Obese**		
Metabolically Healthy	1	1
Metabolically Unhealthy	1.25 (1.01–1.66)	1.16 (0.92–1.46)

OR: Odds ratio; CI: Confidence Interval. Analyses adjusted for age, sex, socioeconomic status, marital status, physical activity, smoking status, alcohol, fruit and vegetable consumption.

^†^Defined as reported physician diagnosis and treatment of any of these three conditions: hypertension, type 2 diabetes and hypercholesterolemia.

Supplementary tables S1 and S2 Tables present the results of analyses with depressive symptoms in three categories. Compared with metabolically healthy normal-weight individuals, the metabolically unhealthy normal-weight, overweight and obese but not the MHO individuals had an increased risk of reporting depressive symptoms more frequently. In analyses stratified by BMI category, the presence of metabolic abnormalities was associated with an increased risk of reporting depressive symptoms. Analyses with a higher threshold of CES-D (S3 and S4 Tables), yielded similar results to those reported in Tables 2 and 3. Sensitivity analyses comparing individuals with and without depression in 1996 did not show any difference for age, sex or BMI but individuals suffering from depression had a higher prevalence of cardio metabolic conditions (results not shown).

## Discussion

In this prospective study of more than 14 000 participants, metabolically healthy obese individuals were at similar risk of depression at the start of follow-up as the metabolically healthy normal weight participants. Over time, risk of depressive symptoms decreased similarly in these two groups. The metabolically unhealthy obese persons were at increased risk of depressive symptoms at start of follow-up, compared to metabolically healthy normal weight persons. Over time, the decrease in depression risk was less marked in metabolically unhealthy obese participants. Compared with metabolically healthy obese individuals, obese participants with metabolic abnormalities had a higher risk of depressive symptoms at start of the follow-up but the decrease in depression risk over time did not significantly differ between these two groups. Overall, metabolic health status rather than obesity predicted depressive symptoms at the start of follow-up, whereas obesity predicted a poorer course of depressive symptoms over time in metabolically unhealthy individuals only.

The prevalence of MHO phenotype in this study is comparable to that in the literature, reported to be between 10% and 50% [25]. To our knowledge, only two studies have examined the risk of depression associated with the MHO phenotype [18,19]. One study conducted on the English Longitudinal Study Ageing (ELSA) cohort found MHO individuals to be at similar risk of depression compared to metabolically healthy non-obese participants after two years of follow up [18]; that study concluded that the association between obesity and the risk of depressive symptoms seemed to be partly dependent on metabolic status. However, a pooled analysis of eight cohorts [19] found an increased risk of depressive symptoms among MHO individuals compared with metabolically healthy non-obese participants and concluded that the phenotype MHO was not a benign condition regarding the risk of depression.

The inconsistency of these results may be due to several factors. First, we assessed metabolic health status based on self-reported data from questionnaires, while in the above-mentioned reports it was based on measured data on clinically assessed blood pressure, triglycerides, HDL-cholesterol, CRP or glycated hemoglobin blood levels. However, this is unlikely to have unduly affected our results as a previous study has shown strong correlations between measured and self-reported weight and height [26]. Secondly, the duration of follow-up was either short in previous analyses [18] or analyses were cross-sectional [19]; our results are based on longitudinal assessment of depressive symptoms over 16 years.

Overall, depressive symptoms at baseline were more closely associated with metabolic health status than obesity. As described in detail previously [18], the mechanisms underlying this association remain unclear; the role of the hypothalamic-pituitary-adrenal axis and the sympathetic nervous system which are associated with stress regulation could play a role. Disturbances of these axes have been associated with depressive symptoms [27] and are likely to be related to insulin resistance and related changes that are observed in the presence of the metabolic syndrome [28,29]. Hamer et al. [18], reported that depression could also result from biochemical changes directly caused by the disruption of metabolic abnormalities and that brain abnormalities, such as reduced white matter and enlarged cerebrospinal fluid space have been reported in obese adolescents with type 2 diabetes, possible due to subtle vascular changes and abnormalities in blood glucose [30]. Finally, observational data even when drawn from a longitudinal study cannot rule out reverse causality or residual confounding. Thus, the association between abnormal metabolic status and depression could be due to unmeasured hazardous behaviors such as poor diet or medical adherence [20,31].

The main strengths of this study include a large sample size, longitudinal design with repeated measurements of BMI, metabolic risk factors and depression data, and a long follow-up period of nearly 16 years. We were also able to take into account several confounders. However, this study also has some limitations. First, since the data were self-reported, participants may have underestimated their weight and overestimated their height that would have the effect of lowering their BMI. Second, depressive symptoms may also be under-reported although previous analyses show that participants of the Gazel cohort reported similar levels of depressive symptoms as the French general population [32]. Third, we were not able to exclude participants with depressive symptoms occurred in the time window when exposure and covariates were measured (1990–1996) because depressive symptoms were assessed for the first time only in 1996. Finally, we assessed depression by the CES-D (23) which does not allow a clinical diagnosis of major depression. However, it is a widely used instrument for the identification of depressive symptoms, which has been shown to be reliable and valid across varied cultural and sociodemographic settings.

To our knowledge, this study is the first prospective study using repeated CES-D measures to examine the associations between the MHO phenotype and depressive symptoms. In summary, our data suggest that the metabolically healthy obese phenotype is not associated with higher risk of depressive symptoms. Poor metabolic health is associated with higher risk of depression, irrespective of BMI. However, when an adverse metabolic profile is combined with obesity it is also associated with poorer longitudinal outcomes for depression.

## Supporting information

S1 Table The association of BMI-metabolic status^†^ phenotypes (1990–96) with depressive symptoms in 3 categories (1996/2012). OR: Odds ratio; CI: Confidence Interval.
^†^Defined as reported physician diagnosis and treatment of any of these three conditions: hypertension, type 2 diabetes and hypercholesterolemia.
Analyses adjusted for age, sex, socioeconomic status, marital status, physical activity, smoking status, alcohol, fruit and vegetable consumption.
(DOCX)

S2 Table The association of metabolic health status^†^ (1990/96) with depressive symptoms in 3 classes in analyses stratified by BMI categories. OR: Odds ratio; CI: Confidence Interval.
^†^Defined as reported physician diagnosis and treatment of any of these three conditions: hypertension, type 2 diabetes, and hypercholesterolemia.
Analyses adjusted for age, sex, socioeconomic status, marital status, physical activity, smoking status, alcohol, fruit and vegetable consumption.
(DOCX)

S3 Table The association of BMI-metabolic status^†^ phenotypes in 1990–96 with depressive symptoms (CESD≥ 23) in 1996/2012. OR: Odds-ratio; CI: Confidence Interval.
^†^Defined as reported physician diagnosis and treatment of any of these three conditions: hypertension, type 2 diabetes, and hypercholesterolemia.
Analyses adjusted for age, sex, socioeconomic status, marital status, physical activity, smoking status, alcohol, fruit and vegetable consumption.
(DOCX)

S4 Table The association of metabolic health status^†^ (1990/96) with depressive (CESD≥ 23) symptoms in analyses stratified by BMI categories. OR: Odds ratio; CI: Confidence Interval.
^†^Defined as reported physician diagnosis and treatment of any of these three conditions: hypertension, type 2 diabetes, and hypercholesterolemia.
Analyses adjusted for age, sex, socioeconomic status, marital status, physical activity, smoking status, alcohol, fruit and vegetable consumption.
(DOCX)
