# Supplementary material for: Metabolically healthy obesity and depressive symptoms: 16-year follow-up of the Gazel cohort study
Source: PLoS One. 2017 Apr 6;12(4):e0174678. doi: 10.1371/journal.pone.0174678 (PMC5383223; doi:10.1371/journal.pone.0174678)
Supplement: S1 Table — OR: Odds ratio; CI: Confidence Interval. †Defined as reported physician diagnosis and treatment of any of these three conditions: hypertension, type 2 diabetes and hypercholesterolemia. Analyses adjusted for age, sex, socioeconomic status, marital status, physical activity, smoking status, alcohol, fruit and vegetable consumption. (DOCX) [file pone.0174678.s001.docx]

Table S1: The association of BMI-metabolic status^†^ phenotypes (1990-96) with depressive symptoms in 3 categories (1996/2012)

|  | **Depressive episodes (1996/2012)** | |
| --- | --- | --- |
|  | **1-3 times vs 0**  **OR (95% CI)** | **4-6 times vs 0**  **OR (95% CI)** |
| **BMI-metabolic health** **status**  Metabolically Healthy-Normal Weight  Metabolically Healthy-Overweight  Metabolically Healthy-Obese  Metabolically Unhealthy-Normal Weight  Metabolically Unhealthy-Overweight  Metabolically Unhealthy-Obese | 1  1.06 (0.95-1.17)  0.96 (0.74-1.25)  1.30 (1.16-1.44)  1.32 (1.19-1.47)  1.50 (1.24-1.82) | 1  0.87 (0.73-1.02)  1.07 (0.74-1.56)  1.48 (1.28-1.72)  1.69 (1.45-1.96)  1.70 (1.30-2.22) |

OR: Odds ratio; CI: Confidence Interval

^†^Defined as reported physician diagnosis and treatment of any of these three conditions: hypertension, type 2 diabetes and hypercholesterolemia.

Analyses adjusted for age, sex, socioeconomic status, marital status, physical activity, smoking status, alcohol, fruit and vegetable consumption.
