# Supplementary material for: Metabolically healthy obesity and depressive symptoms: 16-year follow-up of the Gazel cohort study
Source: PLoS One. 2017 Apr 6;12(4):e0174678. doi: 10.1371/journal.pone.0174678 (PMC5383223; doi:10.1371/journal.pone.0174678)
Supplement: S2 Table — OR: Odds ratio; CI: Confidence Interval. †Defined as reported physician diagnosis and treatment of any of these three conditions: hypertension, type 2 diabetes, and hypercholesterolemia. Analyses adjusted for age, sex, socioeconomic status, marital status, physical activity, smoking status, alcohol, fruit and vegetable consumption. (DOCX) [file pone.0174678.s002.docx]

Table S2: The association of metabolic health status^†^ (1990/96) with depressive symptoms in 3 classes in analyses stratified by BMI categories.

|  | **Episodes de dépression (1996/2012)** | |
| --- | --- | --- |
|  | **1-3 times vs 0**  **OR (95% CI)** | **4-6 times vs 0**  **OR (95% CI)** |
| **Normal weight**  Metabolically healthy  Metabolically unhealthy | 1  1.30 (1.16-1.45) | 1  1.47 (1.27-1.71) |
| **Overweight**  Metabolically healthy  Metabolically unhealthy | 1  1.25 (1.12-1.39) | 1  1.97 (1.66-2.34) |
| **Obese**  Metabolically healthy  Metabolically unhealthy | 1  1.60 (1.18-2.21) | 1  1.61 (1.03-2.54) |

OR: Odds ratio; CI: Confidence Interval

^†^Defined as reported physician diagnosis and treatment of any of these three conditions: hypertension, type 2 diabetes, and hypercholesterolemia.

Analyses adjusted for age, sex, socioeconomic status, marital status, physical activity, smoking status, alcohol, fruit and vegetable consumption.
