# Supplementary material for: Metabolically healthy obesity and depressive symptoms: 16-year follow-up of the Gazel cohort study
Source: PLoS One. 2017 Apr 6;12(4):e0174678. doi: 10.1371/journal.pone.0174678 (PMC5383223; doi:10.1371/journal.pone.0174678)
Supplement: S3 Table — OR: Odds-ratio; CI: Confidence Interval. †Defined as reported physician diagnosis and treatment of any of these three conditions: hypertension, type 2 diabetes, and hypercholesterolemia. Analyses adjusted for age, sex, socioeconomic status, marital status, physical activity, smoking status, alcohol, fruit and vegetable consumption. (DOCX) [file pone.0174678.s003.docx]

Table S3: The association of BMI-metabolic status^†^ phenotypes in 1990-96 with depressive symptoms (CESD≥ 23) in 1996/2012

|  | **At baseline**  **OR (**95% CI**)** | **10-year Change over the follow-up**  **OR (**95% CI**)** |
| --- | --- | --- |
| **Time** | - | 0.52 (0.50-0.55) |
| **BMI-metabolic** health **status**  Metabolically Healthy-Normal Weight  Metabolically Healthy-Overweight  Metabolically Healthy-Obese  Metabolically Unhealthy-Normal Weight  Metabolically Unhealthy-Overweight  Metabolically Unhealthy-Obese | 1 (ref)  0.90 (0.79-1.03)  0.99 (0.73-1.35)  1.33 (1.18-1.49)  1.40 (1.24-1.58)  1.38 (1.12-1.70) | 1 (ref)  1.07 (0.95-1.20)  1.11 (0.97-1.69)  1.01 (0.91-1.13)  0.97 (0.86-1.08)  1.27 (1.06-1.52) |

OR: Odds-ratio; CI: Confidence Interval

^†^Defined as reported physician diagnosis and treatment of any of these three conditions: hypertension, type 2 diabetes, and hypercholesterolemia.

Analyses adjusted for age, sex, socioeconomic status, marital status, physical activity, smoking status, alcohol, fruit and vegetable consumption.
