# Supplementary material for: Metabolically healthy obesity and depressive symptoms: 16-year follow-up of the Gazel cohort study
Source: PLoS One. 2017 Apr 6;12(4):e0174678. doi: 10.1371/journal.pone.0174678 (PMC5383223; doi:10.1371/journal.pone.0174678)
Supplement: S4 Table — OR: Odds ratio; CI: Confidence Interval. †Defined as reported physician diagnosis and treatment of any of these three conditions: hypertension, type 2 diabetes, and hypercholesterolemia. Analyses adjusted for age, sex, socioeconomic status, marital status, physical activity, smoking status, alcohol, fruit and vegetable consumption. (DOCX) [file pone.0174678.s004.docx]

Table S4: The association of metabolic health status^†^ (1990/96) with depressive (CESD≥ 23) symptoms in analyses stratified by BMI categories.

|  | **At baseline**  **OR (**95% CI**)** | **10-year Change over the follow-up**  **OR (**95% CI**)** |
| --- | --- | --- |
| **Normal weight**  Metabolically healthy  Metabolically unhealthy | 1  1.33 (1.19-1.50) | 1  1.01 (0.91-1.12) |
| **Overweight**  Metabolically healthy  Metabolically unhealthy | 1  1.56 (1.36-1.79) | 1  0.91 (0.79-1.04) |
| **Obese**  Metabolically healthy  Metabolically unhealthy | 1  1.33 (1.03-1.92) | 1  1.01 (0.74-1.37) |

OR: Odds ratio; CI: Confidence Interval.

^†^Defined as reported physician diagnosis and treatment of any of these three conditions: hypertension, type 2 diabetes, and hypercholesterolemia.

Analyses adjusted for age, sex, socioeconomic status, marital status, physical activity, smoking status, alcohol, fruit and vegetable consumption.
